# Role of Hippo Pathway-YAP/TAZ Signaling in Angiogenesis

**DOI:** 10.3389/fcell.2019.00049

**Published:** 2019-04-10

**Authors:** Gandhi T. K. Boopathy, Wanjin Hong

**Affiliations:** Institute of Molecular and Cell Biology, Agency for Science, Technology and Research, Singapore, Singapore

**Keywords:** Hippo pathway, YAP, TAZ, angiogenesis, signal transduction, mammalian Hippo signaling

## Abstract

Angiogenesis is a highly coordinated process of formation of new blood vessels from pre-existing blood vessels. The process of development of the proper vascular network is a complex process that is crucial for the vertebrate development. Several studies have defined essential roles of Hippo pathway-YAP/TAZ in organ size control, tissue regeneration, and self-renewal. Thus Hippo pathway is one of the central components in tissue homeostasis. There are mounting evidences on the eminence of Hippo pathway-YAP/TAZ in angiogenesis in multiple model organisms. Hippo pathway-YAP/TAZ is now demonstrated to regulate endothelial cell proliferation, migration and survival; subsequently regulating vascular sprouting, vascular barrier formation, and vascular remodeling. Major intracellular signaling programs that regulate angiogenesis concomitantly activate YAP/TAZ to regulate key events in angiogenesis. In this review, we provide a brief overview of the recent findings in the Hippo pathway and YAP/TAZ signaling in angiogenesis.

## Introduction

The Hippo signaling pathway is an evolutionarily conserved signaling pathway that is the latest addition to the family of signaling pathways known to be involved in control organ size and development (Pan, 2010; Halder and Johnson, 2011). The Hippo pathway is an evolutionarily conserved serine/threonine kinase signaling cascade originally identified in fruit fly (*Drosophila melanogaster*) (Harvey et al., 2003; Wu et al., 2003). This discovery of a new signaling pathway is a key advance in the understanding of the signaling networks controlling metazoan physiology and development. A list of key Hippo pathway genes in fruit fly and the orthologs in humans are listed in Table 1. Hippo pathway negatively regulates the activity of transcriptional co-activators, Yes-associated protein 1 (YAP) and Transcriptional coactivator with PDZ-binding motif (TAZ); both YAP and TAZ are orthologs of *Drosophila* Yorkie [*Yki*]. YAP and TAZ are transcriptional coactivators, do not have DNA binding capacity. So, active YAP/TAZ translocate to nucleus and interact majorly with TEA domain family member (TEAD) transcription factors (TEAD1-4; orthologs of *Drosophila* Scalloped [*Sd*]). In nucleus, the YAP/TAZ-TEAD protein complex transcribes genes that control cell proliferation, apoptosis and cell fate (Varelas, 2014).

**Table 1:** List of key Hippo pathway genes in fruit fly (*Drosophila melanogaster*) and the respective orthologs in humans.

Human protein name	Human *gene name*	*Drosophila* protein (*gene name*)
Mammalian STE20-like kinase 1 (MST1) Mammalian STE20-like kinase 2 (MST2)	*STK4* *STK3*	Hippo (*Hpo*)
Neurofibromin-2/Merlin	*NF2*	Merlin (*Mer*)
MOB kinase activator 1A MOB Kinase Activator 1B	*MOB1A* *MOB1B*	Mats (*Mats*)
Salvador homolog 1	*SAV1*	Salvador (*Sav*)
Large tumor suppressor kinase 1 Large tumor suppressor kinase 2	*LATS1* *LATS2*	Warts (*Wts*)
Yes-associated protein 1/ YAP WW Domain Containing Transcription Regulator 1/TAZ	*YAP1* *WWTR1*	Yorkie (*Yki*)
TEA domain family member 1 TEA domain family member 2 TEA domain family member 3 TEA domain family member 4	*TEAD1* *TEAD2* *TEAD3* *TEAD4*	Scalloped (*Sd*)
Kidney and brain expressed protein (Kibra)	*WWC1*	Kibra (*Kibra*)
Crumbs homolog 1 (Crumbs)	*CRB1*	Crumbs (*Crb*)

Angiogenesis is an organized process of formation of new blood vessels from pre-existing vessels by involving a series of events that comprise endothelial cell sprouting, branching, formation of lumen, and remodeling into a functionally perfused vascular network (Chung and Ferrara, 2011; Potente et al., 2011). Angiogenesis is the fundamental to multiple biological processes such as development, wound healing, and reproduction (Carmeliet and Jain, 2011). Dysregulation of angiogenesis is a key factor in the progression of a variety of diseases including diabetic retinopathy, age-related macular degeneration (AMD), rheumatoid arthritis and cancer (Aiello et al., 1994; Paleolog, 2002; Carmeliet, 2003; Nishida et al., 2006; Crawford et al., 2009; Ambati and Fowler, 2012). Recently, a wealth of studies has significantly expanded the role of Hippo pathway network in angiogenesis. The aim of this review is to outline the current findings in Hippo pathway-YAP/TAZ in angiogenesis.

## Hippo-YAP/TAZ Pathway

**FIGURE 1:**
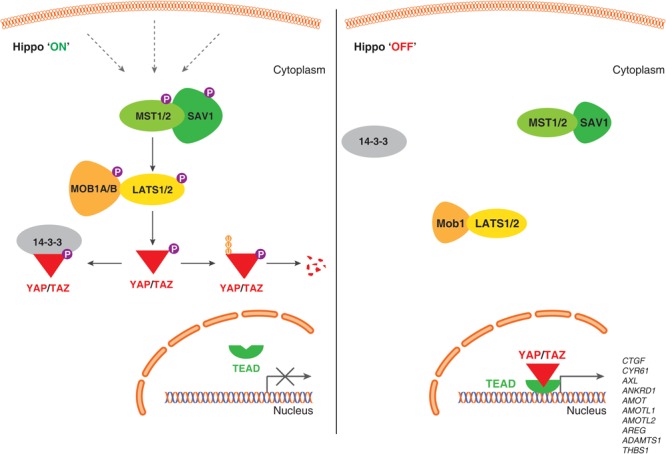
Schematic representation of the Hippo signaling pathway-YAP/TAZ in human endothelial cells. When the Hippo signaling pathway is active/on **(Left)**, multiple upstream signals regulate the phosphorylation of MST1/MST2, LATS1/LATS2 kinases, and phosphorylates YAP/TAZ proteins. Phosphorylation of YAP/TAZ recruits 14-3-3 proteins that stimulate cytoplasmic retention or proteolytic degradation. When Hippo signaling pathway is inactive/off **(Right)**, YAP/TAZ are not phosphorylated, localize to the nucleus, form a complex with transcription factor TEADs and regulate genes required for endothelial cell proliferation, migration and survival.

In mammals, the core Hippo pathway is largely characterized by Serine/Threonine kinases; mammalian Sterile 20-related 1 and 2 kinases (MST1 and MST2; orthologs of *Drosophila* Hippo [*Hpo*]) and Large tumor suppressor 1 and 2 kinases (LATS1 and LATS2; orthologs of *Drosophila* Warts [*Wts*]) (Figure 1).

MST kinases are known to undergo auto-activation through auto-phosphorylation on the activation loop of the MST dimer, MST1 at Thr183 and MST2 at Thr180 (Deng et al., 2003; Praskova et al., 2004). The carboxyl terminus of MST kinases have a distinctive coiled-coil structure called SARAH domain [named after the three genes that contain the homologous structures – Salvador (Salvador 1/WW45), RASSF1-6 and Hippo (MST1/MST2) (Scheel and Hofmann, 2003)]. SARAH domain mediates homo- and heterodimerization of MST1/MST2. MST1/MST2 heterodimer forms a complex with SARAH domain containing protein, Salvador 1 (SAV1) (Callus et al., 2006). Being 96% identical proteins, MOB kinase activator 1A and 1B (MOB1A and MOB1B) forms a complex with LATS1 and LATS2 kinases. MST1/MST2 kinases activate LATS1 and LATS2 kinases by phosphorylating at Thr1079 and Thr1041, respectively (Chan et al., 2005). Both MOB1A and MOB1B are phosphorylated by MST1/MST2 kinases at Thr35 and Thr12; these phosphorylation events promote the interaction of MOB1A/MOB1B to LATS1/LATS2 (Praskova et al., 2008). Also, phosphorylation at MOB1A/MOB1B promotes its affinity to LATS and this interaction leads to the auto-phosphorylation of LATS1/LATS2 at the activation loop (Ser909 at LATS1 and Ser872 at LATS2) (Chan et al., 2005; Hergovich et al., 2006a; Praskova et al., 2008). Both, phosphorylation by MST1/MST2 kinases and the auto-phosphorylation on LATS are critically required for LATS1/LATS2 kinase activation (Galan and Avruch, 2016).

Large tumor suppressor kinases belong to AGC group of kinases (named after the protein kinase A, G, and C families) that recognize the substrate consensus sequence HXRXXS/T (Mah et al., 2005; Hergovich et al., 2006b). Key substrates of LATS kinases are transcription co-activators, YAP and TAZ. YAP is a key transcriptional regulator and the first protein identified with a WW domain (a motif comprising of two Tryptophan [W] residues) (Sudol, 1994; Sudol et al., 1995) and TAZ is a YAP paralog (with 44% identity to YAP). YAP has five (Ser61, Ser109, Ser127, Ser164, and Ser381) and TAZ has four (Ser66, Ser89, Ser117, and Ser311) HXRXXS/T motifs phosphorylated by LATS1/LATS2 kinases (Zhao et al., 2007; Hao et al., 2008; Lei et al., 2008). Phosphorylation of YAP and TAZ by LATS kinases either primes to their binding with 14-3-3 proteins leading to cytoplasmic sequestration of YAP/TAZ (Zhao et al., 2007; Lei et al., 2008) or ubiquitin-mediated protein degradation (Zhao et al., 2010; Figure 1).

When LATS1/LATS2 kinases are inactive, YAP/TAZ are not phosphorylated and translocate to nucleus. YAP/TAZ do not comprise DNA binding domain, but they bind to TEAD transcription factor family (TEAD1 – 4) to mediate target gene expression such as connective tissue growth factor (CTGF), cysteine-rich angiogenic inducer 61 (CYR61) and others to promote cell growth, proliferation, migration, and survival (Chan et al., 2008; Lei et al., 2008; Zhao et al., 2008, 2010; Pobbati et al., 2012). Also, YAP/TAZ has been shown to interact with a variety of transcription factors, such as SMAD1 (Alarcon et al., 2009), SMAD2/3 (Varelas et al., 2008, 2010), SMAD7 (Ferrigno et al., 2002), RUNT-related transcription factors (RUNX1 and RUNX2) (Yagi et al., 1999), T-box transcription factor 5 (TBX5) (Murakami et al., 2005; Rosenbluh et al., 2012), and p73 (Strano et al., 2001). Also, YAP is shown to interact with the intracellular domain (ICD) of ErbB4 (one of the members of the epidermal growth factor receptors) in the nucleus (Haskins et al., 2014) to mediate modulate the expression of genes involved in proliferation, differentiation, and development.

Loss of any of the core components such as Merlin, MST1/MST2, Salvador-1, LATS1/2, MOB1A/MOB1B show an upregulation of YAP/TAZ-TEAD target gene transcription such as cell proliferation and tissue growth – justifying roles of these core members in the Hippo pathway (Camargo et al., 2007; Dong et al., 2007; Zhou et al., 2009; Lee et al., 2010; Zhang et al., 2010; Nishio et al., 2012).

### Regulation of the Hippo Signaling Pathway

**FIGURE 2:**
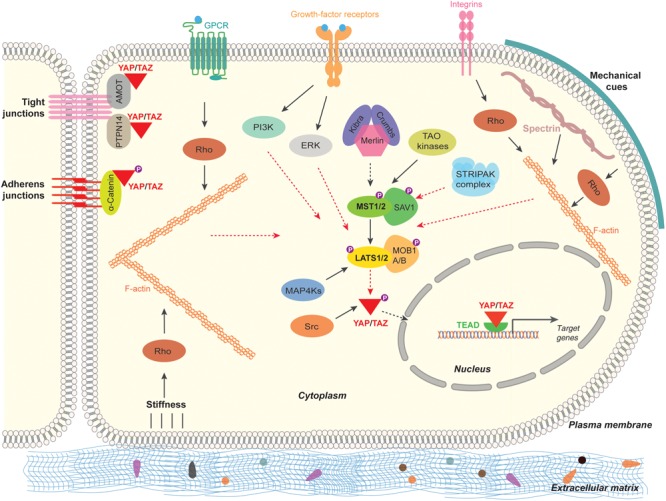
Regulation of the Hippo pathway-YAP/TAZ in mammalian cells. The Hippo signaling pathway-YAP/TAZ in mammals are regulated by multitude of signaling mechanisms. Cell polarity and cell adhesion proteins (α-Catenin, PTPN14, AMOT), mechanical signals (stiffness caused by ECM, cell contact and cell geometry) and signal transduction (GPCR, growth-factor-receptor, Integrins, Src, MAP4Ks, TAO kinases, STRIPAK complex). Black arrow lines indicates activation and the red arrow dashed lines indicate inhibition.

The central mechanism to regulate the transcriptional co-activators – YAP/TAZ is by influencing the subcellular localization and the protein stability through phosphorylation by upstream kinases such as LATS1/LATS2 kinases (Zhao et al., 2007, 2010). Numerous studies have shown that multitude of intracellular, extracellular, and non-cellular mechanisms can activate Hippo pathway and regulate YAP/TAZ activity (Figure 2). Following are the brief cellular events that regulate Hippo pathway and YAP/TAZ activity:

### Hippo Pathway-YAP/TAZ Regulation by Cell Polarity and Cell Adhesion

Generally cells exhibit polarity. Polarity is a unique asymmetric organization of cellular components to carry out specified functions (Bryant and Mostov, 2008). Intercellular junctions such as adherens junctions (AJ), tight junctions (TJ), and gap junctions mediate communication and adhesion between adjoining epithelial and endothelial cells (Bazzoni and Dejana, 2004). Apical and basolateral cell polarity is established by AJs and TJs with the help of well-defined proteins in complexes called as polarity complexes (Assemat et al., 2008; Martin-Belmonte and Perez-Moreno, 2011). Crumbs (CRB1) is a transmembrane protein solely localized in the apical plasma membrane and is essential for substantiating the apical-basal polarity of a cell (Tepass et al., 1990). Crumbs in association with FERM domain-containing protein 6 (FRMD6; Expanded Homolog), Kibra (WW domain containing protein) and Merlin (also called Neurofibromin 2 or Schwannomin) interacts and activates the Hippo pathway (Hamaratoglu et al., 2006; Baumgartner et al., 2010; Ling et al., 2010; Robinson et al., 2010; Yu et al., 2010).

Angiomotin family of proteins consists of three proteins, angiomotin (AMOT), angiomotin-like 1 (AMOTL1), and angiomotin-like 2 (AMOTL2). They are characterized by coiled-coil domain, WW binding motif, actin binding domain, and PDZ-binding domain (Bratt et al., 2002, 2005). Through these domains, AMOTs localize to TJs, acts as a scaffold protein bind to actin cytoskeleton, YAP/TAZ proteins and Rho-GTPases. AMOTs directly bind and negatively regulate the localization and the activity of YAP and TAZ both dependent and independent of Hippo pathway (Chan et al., 2011; Wang et al., 2011; Zhao et al., 2011).

Cadherins are the key components of AJ that bind cells with each other. α-Catenin connect between cadherins and actin cytoskeleton in cells. α-Catenin forms a complex with 14-3-3 proteins and phosphorylated YAP and suppresses YAP activity by retaining YAP in the cytoplasm causing inactivation of YAP (Kim et al., 2011; Schlegelmilch et al., 2011). Also, another component of AJ, Protein-tyrosine phosphatase type 14 (PTPN14) interacts with YAP through its PPXY motif and the WW domain of YAP. This interaction retains YAP to cytoplasmic front and causes inactivation of YAP (Wang et al., 2012; Liu et al., 2013). By interfering the function of AJs or TJs cause an activation of YAP/TAZ in multiple cells types (Varelas et al., 2010).

### Hippo Pathway-YAP/TAZ Regulation by Mechanical Cues

Mechanical forces such as stress, strain or distortion physiologically impacting cell density, stiffness of the extracellular environment and cell geometry are known to regulate the localization and activity of YAP/TAZ (Zhao et al., 2007, 2012; Dupont et al., 2011). The activity of YAP/TAZ in response to mechanical forces depends on the reorganization of actin cytoskeleton and the activity of Rho GTPases (Aragona et al., 2013; Reginensi et al., 2013). Also, Spectrin – one of the cytoskeletal proteins play a key role in the maintenance of plasma membrane integrity (Machnicka et al., 2014). Spectrin by associating with actin filaments play an important role in the activity of YAP as the interruption of the spectrin cytoskeletal network is shown to activate YAP in fruit fly and humans (Deng et al., 2015; Fletcher et al., 2015; Wong et al., 2015). Also, recently, Roca-Cusachs and colleagues have shown that application of force directly to the nucleus is sufficient for the nuclear accumulation of YAP. Force transmission cause flattening of nucleus, stretches nuclear pore complexes (NPC), this causes mechanical resistance to molecular transport across NPC and resulting in nuclear import of YAP (Elosegui-Artola et al., 2017).

### Hippo Pathway-YAP/TAZ Regulation by Cellular Signal Transduction

Several studies have shown that multiple extracellular ligands/growth factors and multiple signaling pathways regulate Hippo pathway. Earlier, ligands of G-protein-coupled receptors (GPCRs) such as lysophosphatidic acid (LPA) and sphingosine 1-phosphate (S1P) after association with GPCR, signals through G-proteins (Gα12/13) inhibits LATS1/LATS2 thereby activating YAP/TAZ (Yu et al., 2012).

Epidermal growth factor (EGF) through EGF-receptor (EGFR) inactivates Hippo pathway through phosphoinositide 3-kinase (PI3K) and phosphoinositide-dependent kinase (PDK1) independent of AKT signaling (Fan et al., 2013). Also, EGFR is known to activate YAP/TAZ through Ras-Raf-Mitogen-activated protein kinases (MAPK) signaling axis (Reddy and Irvine, 2013). Besides EGF, amphiregulin (AREG) is a growth factor that activates EGFR and in-turn activate YAP/TAZ (Zhang et al., 2009). Interestingly, AREG is a transcriptional target of YAP/TAZ (Zhang et al., 2009) Focal adhesions (FAs) in cells are strongly associated with the extracellular matrix (ECM). Through specific ligands or through mechanical cues, integrins in FAs activate YAP/TAZ through Rho-GTPases (Tang et al., 2013; Elbediwy et al., 2016; Varzavand et al., 2016). Integrins are also known to signal through Src family kinases to regulate YAP/TAZ (Kim and Gumbiner, 2015; Elbediwy et al., 2016). Mitogen-activated protein kinase kinase kinase kinases (MAP4K), MAP4K1/2/3/5 phosphorylate and activate Hippo pathway core component LATS1/LATS2 and inactivates YAP/TAZ (Meng et al., 2015; Zheng et al., 2015). It appears that MST1/MST2 and MAP4Ks could have partially redundant roles in regulating LATS1/2 kinases (Meng et al., 2015, 2016). Src can phosphorylate activate YAP both by direct phosphorylation on the tyrosine residues (Y341, Y357, and Y394) and through inhibiting LATS1/LATS2 by phosphorylation (Kim and Gumbiner, 2015; Li et al., 2016; Si et al., 2017). Interestingly, integrins also inactivate Hippo signaling to activate YAP/TAZ by inactivating Merlin through integrin-linked kinase (ILK) (Serrano et al., 2013).

Other kinases, such as thousand-and-one amino acids kinases (TAOK1/2/3) have been shown to directly phosphorylate and activate Hippo pathway core component MST1/MST2 kinases (Boggiano et al., 2011; Poon et al., 2011). In striatin-interacting phosphatase and kinase (STRIPAK) complex, the core of STRIPAK complex is the phosphatase protein phosphatase 2A (PP2A). PP2A is known to interact and dephosphorylate MST1/MST2 kinases causing the activation of YAP/TAZ (Couzens et al., 2013; Zheng et al., 2017).

## Hippo Pathway in Angiogenesis

Studies have shown that YAP/TAZ expression was elevated in the retinal blood vasculature at an early stage of development (from postnatal day 5) and during differentiation of endothelial progenitor cells to matured endothelial cells in mice (Choi et al., 2015; Neto et al., 2018). Similar to the observation in multiple cell types, YAP is inactivated by phosphorylation and redistributed in a cell contact-dependent manner in human umbilical vein endothelial cells (HUVEC). In HUVECs, contact-inhibition is mainly due to vascular endothelial cadherin (VE-cadherin)-mediated cell junctional complex regulation (Choi et al., 2015). Knockdown of YAP in HUVECs and mice aortic rings showed a significant decrease in the total tubular network and endothelial sprouts, respectively (Choi et al., 2015). Similarly, endothelial cell-specific loss of YAP/TAZ in mice results in embryonic lethality (Choi et al., 2015; Kim et al., 2017; Wang et al., 2017) – clearly indicating YAP/TAZ as a critical regulator for vascular sprouting, vascular barrier formation and angiogenesis (Kim et al., 2017).

### YAP/TAZ in VEGF Signaling

Vascularization is an important process during tissue development which is accurately mediated by vascular endothelial growth factor A (VEGF) (Coultas et al., 2005). VEGF is a primary factor for the initiation of sprouting angiogenesis and major regulator of blood vessel formation (Chappell et al., 2011). VEGF induces angiogenesis by associating with VEGF receptors VEGFR1 and VEGFR2 through endothelial cell proliferation, survival and migration (Carmeliet, 2005; Cebe-Suarez et al., 2006). During angiogenesis, VEGF-VEGFR2 signaling-axis critically requires YAP/TAZ activity to mediate angiogenesis (Kim et al., 2017; Wang et al., 2017). Activation of YAP/TAZ upon VEGF stimulation involves the activation Src family kinases and successive cytoskeletal remodeling in endothelial cells (Kim et al., 2017; Wang et al., 2017). Using bioluminescence-based biosensor of LATS kinase activity discovered that VEGF/VEGFR signaling pathway activates PI3K-AKT and MEK-ERK signaling axes that leads to the inhibition of MST kinases (MST1/MST2) and LATS kinases (LATS1/LATS2) and consecutive activation of YAP/TAZ (Azad et al., 2018). Clearly, YAP/TAZ are the key components of VEGF signaling in angiogenesis.

YAP/TAZ are also known to regulate VEGF signaling pathway. In HUVECs, siRNA based knockdown of YAP/TAZ or inhibition of YAP/TAZ by verteporfin effectively reduced VEGF-induced angiogenesis (Azad et al., 2018). Also, administration of verteporfin significantly reduced VEGF-induced blood vessel formation in Matrigel plugs using mouse endothelial cells (Azad et al., 2018), suggesting an important role of YAP/TAZ in VEGF-induced angiogenesis *in vivo*.

Also, activation of VEGF-VEGFR2-Src signaling axis also promotes YAP/TAZ based expression of several cytoskeletal reorganization genes such as Myosin 1C (MYO1C) (Wang et al., 2017). MYO1C is one of nine isoforms of the myosin 1 gene in humans (Gillespie and Cyr, 2004). The cell surface distribution of VEGFR2 is regulated by MYO1C in endothelial cells (Tiwari et al., 2013). During angiogenesis, as a positive feedback loop, YAP/TAZ could regulate VEGF induced signal transduction by regulating VEGFR2 localization to the cell surface through the regulation of MYO1C.

### YAP/TAZ in Angiopoietin-2/Tie2 Signaling

Angiopoietins are a family of growth factors that include angiopoietins 1–4 (Ang1–4) and the receptors for angiopoietins are tyrosine kinases include Tie receptors (Tie1 and Tie2). Tie1 and Tie2 receptors are predominantly expressed in endothelial cells (Fagiani and Christofori, 2013). Angiopoietins/Tie signaling axis has been suggested to play a critical role in sprouting angiogenesis and vascular remodeling (Hanahan, 1997). Ang2 is an autocrine factor highly secreted by endothelial cells at sites of active vascular remodeling and regulates angiogenesis in a pro-angiogenic or anti-angiogenic manner (Maisonpierre et al., 1997; Daly et al., 2006). In HUVECs and during post-natal development of the mouse retina, Ang2 is identified as a key target gene of YAP in endothelial cells; regulation of angiogenesis and vascular remodeling by YAP is shown to mediated by Ang2 (Choi et al., 2015; Azad et al., 2018).

### YAP/TAZ in Other Signaling Pathways/Events

The bona fide YAP/TAZ target genes are connective tissue growth factor (CTGF) and cysteine-rich angiogenic inducer 61 (CYR61) (Zhao et al., 2008). CYR61 and CTGF are CCN family (CYR61, CTGF, and NOV proteins) of extracellular matrix associated angiogenic regulators with 47% amino acid sequence identity (Lau and Lam, 1999). Both, CTGF and CYR61 bind to a variety of cell surface receptors in a context-dependent manner, including integrin receptors and exert endothelial cell function and angiogenesis (Brigstock, 2002). CYR61 and CTGF are the well-known ligands of integrins αVβ3, αIIbβ3, αvβ5, and α6β1 (Kireeva et al., 1998; Jedsadayanmata et al., 1999; Chen et al., 2001; Grzeszkiewicz et al., 2001). CTGF and CYR61 are shown to induce angiogenesis, at least in part, through the activation of integrin αVβ3 pathway and also involved in tumor angiogenesis (Babic et al., 1998; Grzeszkiewicz et al., 2001).

YAP interacts with signal transducer and activator of transcription 3 (STAT3) in endothelial cells and this interaction maintains STAT3 in the nucleus and increases the transcription of ANG2 thereby promotes angiogenesis and vessel remodeling (He et al., 2018). Mochizuki and colleagues observed that YAP translocate into the nucleus after the onset of blood-flow and relocated to cytoplasm after the flow was interrupted in endothelial cells using transgenic zebrafish (Nakajima et al., 2017).

### YAP/TAZ as Mechanical Sensors of Endothelium

Endothelial cells can sense mechanical forces, trigger cellular signaling and result in cell proliferation (Liu et al., 2007). Using human lung microvascular endothelial cells (HMVEC), Piccolo’s group have showed that cytoskeletal adaptation to the cell spread causes activation and nuclear accumulation of YAP/TAZ and when cells are maneuvered into a compact and round shape YAP/TAZ are inactivated and localized in the cytoplasm (Dupont et al., 2011). Also, Gerhardt’s group have shown that the cellular stretch have increased the cell proliferation of HUVEC to fivefold which is dependent on YAP in response to mechanical stimulation at cell–cell junctions by VE-Cadherin (Neto et al., 2018). Interestingly, shear force caused by the blood-flow has also been shown to transport YAP/TAZ to nucleus in endothelial cells (Lai and Stainier, 2017; Nagasawa-Masuda and Terai, 2017; Nakajima et al., 2017). Together, in endothelial cells, YAP/TAZ transduce mechanical signals exerted by the rigidity of the blood-flow, extracellular matrix and the cell geometry. However, the mechanical regulation of YAP/TAZ exists in a variety of cell types, regardless of their epithelial, mesenchymal, or endothelial nature (Piccolo et al., 2014), indicating a common mechanism of mechanical regulation of YAP/TAZ in all these cell types.

### Hippo Pathway-YAP/TAZ in Retinal Angiogenesis

YAP and TAZ have been shown to play a key role in the development of retinal blood vessels. Targeted loss of YAP/TAZ in blood vessels, in early post-natal days, shows a substantially reduced vascular density and vessel outgrowth in retina (Kim et al., 2017). Also, the assembled blood vessels were enlarged (increased vessel diameter) and twisted in YAP/TAZ knockout (Kim et al., 2017). Interestingly, there was no vascular network observed in the deep vascular plexus, reduction of tight junction proteins (ZO1 and Claudin-5) and disordered vascular endothelial cadherin (VE-cadherin) were found in the retinas with the loss of YAP/TAZ (Kim et al., 2017). Also, YAP/TAZ is involved in the formation and maturation of the blood-retinal barrier (BRB) and blood-brain barrier (BBB) but not required for maintaining blood-barrier integrity during adulthood (Kim et al., 2017). YAP and TAZ are shown to be involved in junctional remodeling in endothelial cell and also, inhibited Notch and BMP signaling events in endothelial cells (Neto et al., 2018).

### Angiogenic Regulation by Hippo Pathway-YAP/TAZ Signaling in Mice and Fish

The vasculature and the basic vascular plan of zebrafish embryos are quite similar to that of other mammalian models (Roman and Weinstein, 2000; Lawson and Weinstein, 2002). Whole-body knockout of *Yap* in mice resulted in developmental arrest around embryonic day 8.5 due to vascular defects (Morin-Kensicki et al., 2006). Upon knockdown of YAP, using specific RNAs, in mouse retina resulted in significantly reduced vascular density (Choi et al., 2015). Endothelial specific loss of YAP in mice results in embryonic lethality because of impaired heart valve development caused by defect in endothelial-to-mesenchymal transition (Zhang et al., 2014). Also, endothelial cell specific knockout of YAP/TAZ results in vascular defects during embryonic and postnatal development (Wang et al., 2017). These results clearly indicate that YAP is required for the early stages in the development of vasculature and placenta of mice. Interestingly, in zebrafish, inhibition or activation of YAP did not yield any significant abnormalities during angiogenesis (Hu et al., 2013; Agarwala et al., 2015; Nagasawa-Masuda and Terai, 2017; Nakajima et al., 2017). Loss of YAP/TAZ results in death of zebrafish due to severe developmental defects earlier than vascular development (Nakajima et al., 2017) making it harder to study the role of YAP/TAZ in developmental vasculature in zebrafish. However, YAP is shown to be playing a key role in the maintenance of blood vessels in zebrafish (Nakajima et al., 2017).

### Vascular Regression and Vessel Retraction

During angiogenesis, vascular networks undergo extensive vascular remodeling, such as vascular pruning and regression to form mature vasculature (Korn and Augustin, 2015). Understanding molecular mechanisms of vessel regression has key therapeutic implications in diseases such as cancer and retinal diseases. Blood-flow has been shown to positively regulate endothelial YAP/TAZ, hence YAP/TAZ may sense the blood flow to regulate vascular shrinking for vascular regression (Nagasawa-Masuda and Terai, 2017; Nakajima et al., 2017). Inhibition of YAP/TAZ-TEAD transcriptional activity disrupts the vascular regression of caudal vein plexus in zebrafish (Nagasawa-Masuda and Terai, 2017). Complete loss of YAP showed vessel thinning and vessel retraction in dorsal longitudinal anastomotic vessel of zebrafish (Nakajima et al., 2017).

### YAP/TAZ in Vascular Diseases

A gradual accumulation of deposits such as fat, cholesterol and cellular debris on the walls of arteries are a key characteristic of atherosclerosis, leading to stroke, or heart attack (Caro et al., 1969; Ross, 1999). In Endothelial cells, YAP/TAZ activity was high in disturbed shear stress (blood flow was disturbed mimicking atherogenic mechanical stress) than a uniform laminar shear stress (atheroprotective) (Wang K.C. et al., 2016; Wang L. et al., 2016). In mice model for atherosclerotic- and human atherosclerotic-blood vessels, very high YAP/TAZ activity was observed (Wang K.C. et al., 2016; Wang L. et al., 2016), indicating mechanotransduction of YAP/TAZ is responsible for pathological effects of disturbed blood flow during atherosclerosis.

Pulmonary hypertension (PH) is a dangerous vascular disease represented by high blood pressure in the pulmonary arteries that can lead to heart failure. PH is characterized by vascular remodeling due to proliferation of smooth muscle cells and endothelial cells (Veyssier-Belot and Cacoub, 1999). After examination of pulmonary endothelial cells from lung tissues of PH patients, ECM stiffening has been shown to mechanoactivate YAP/TAZ, resulting in endothelial cell proliferation and migration, thereby YAP/TAZ advances the pathogenesis of PH (Bertero et al., 2016). These studies clearly suggest that the inhibition of YAP/TAZ activity could be a treatment option for multiple diseases in the future.

## Discussion

**FIGURE 3:**
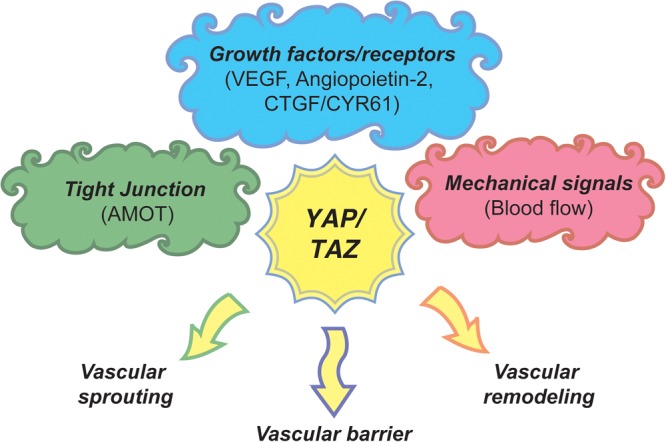
A model of Hippo pathway-YAP/TAZ angiogenic program. Upstream signaling cues such as tight junction proteins, growth factors/receptors and mechanical signals regulate the activity of YAP/TAZ through Hippo pathway or independent mechanisms to regulate endothelial cell proliferation, migration and survival subsequently regulating vascular sprouting, vascular barrier formation, and vascular remodeling.

Research around the past decade has greatly lead to our understanding of the molecular mechanism, cellular and the physiological function of the Hippo signaling pathway. An array of studies have strongly conclusively proved the Hippo pathway as a key mechanism of regulation of organ size and tissue maintenance in metazoa. Angiogenesis is a key biological process of formation of blood vessels that is required for the transportation of required nutrients and oxygen to all parts of the body and brings back unwanted waste from the organs and tissues during health and disease states of an organism. At the organismal level, generation and maintenance of blood vessels during development and wound healing in physiological and pathological conditions is pivotal. Hence, the signaling events pertaining to endothelial cell proliferation, migration, and maintenance has been a major research focus. The inactivation of Hippo signaling pathway and/or activation of YAP/TAZ is vital for the functional consequences of multiple signaling pathways such as VEGF, angiopoietin, CYR61, etc., in angiogenesis. Multiple cell signaling pathways concomitantly activating YAP/TAZ in endothelial cells clearly imply that Hippo pathway-YAP/TAZ is the crucial signaling-nexus in vascular growth, migration, branching, and vascular network maintenance (Figure 3). However, exactly how the Hippo pathway-YAP/TAZ orchestrates such a vast degree of biological processes and the pertaining molecular mechanisms are currently not known. Elucidating the functional aspects of endothelial cell specific target genes of YAP/TAZ would be of utmost importance in understanding the role of Hippo pathway-YAP/TAZ in angiogenesis.

All the recently reported signaling events, MAP kinase signaling, Rho family of GTPases, etc., pertaining to the activation of YAP/TAZ in endothelial cells were reported in multiple other cell types and/or model organisms. However, one of the major questions is, is there any signaling event regulating the activity of YAP/TAZ specifically in endothelial cells? If there is any such event, it would be of great importance for therapeutic interference in diabetic retinopathy, AMD, rheumatoid arthritis and cancers.

It is quite interesting that YAP is essential for developmental angiogenesis in mice but not in zebrafish. However, it is possible that TAZ could take over the essential functions of YAP in the early development of zebrafish or YAP may be a regulating the expression of some essential genes in angiogenesis in mice but not in zebrafish. Further studies on the differences in the gene targets of YAP in mice and zebrafish endothelium could provide an answer.

The components of Hippo pathway and the transcriptional co-activators YAP/TAZ are promising therapeutic targets for multiple ailments (Park and Guan, 2013). Currently, most commonly used anti-angiogenic agents in clinics are against VEGF (Meadows and Hurwitz, 2012). However, the clinical benefits with anti-VEGF therapies either develop resistance (primary or acquired resistance) or quite modest (Ebos et al., 2009; Yang et al., 2016). Neutralizing antibodies against VEGF pathway target as well as YAP/TAZ target genes – CTGF and CYR61 would be a valuable addition to the repertoire of therapeutic antibodies targeting angiogenesis.

Endothelial cells are well known to sense mechanical forces and resulting in cellular responses pertaining to proliferation (Liu et al., 2007). In a key study, Dupont et al. (2011) have showed that the cell geometry plays key role in the proliferation of endothelial cells. Also, shear force caused by the blood-flow has also been shown to activate YAP/TAZ in endothelial cells. Interestingly, this shear flow (disturbed flow due to atherosclerotic plaques) is sensed by the YAP/TAZ in the endothelial cells and are responsible for pathological effects of disturbed blood flow during atherosclerosis and PH. These studies point to a new paradigm inhibition of YAP/TAZ activity in treatment option for multiple diseases in the future.

## Author Contributions

GB and WH researched the relevant literature and conceived and wrote the manuscript.

## Conflict of Interest Statement

The authors declare that the research was conducted in the absence of any commercial or financial relationships that could be construed as a potential conflict of interest.
